# Organoids for transplant research

**DOI:** 10.3389/ti.2026.16963

**Published:** 2026-06-29

**Authors:** Ekaterine Berishvili

**Affiliations:** 1 Laboratory of Tissue Engineering and Organ Regeneration, Department of Surgery, University of Geneva, Geneva, Switzerland; 2 Cell Isolation and Transplantation Center, Department of Surgery, Geneva University Hospitals and University of Geneva, Geneva, Switzerland; 3 Faculty Diabetes Center, University of Geneva Medical Center, Geneva, Switzerland; 4 Institute of Medical and Public Health Research, Ilia State University, Tbilisi, Georgia

**Keywords:** cholangiocyte organoids, kidney organoid, normothermic machine perfusion, organoid, pancreatic organoid

Within little more than a decade, organoid technology has progressed from a striking demonstration of cellular self-organisation to one of the most generative tools in regenerative medicine. The observation that single Lgr5^+^ intestinal stem cells could rebuild crypt–villus structures without a mesenchymal niche [1] opened a path that has since yielded kidney, liver, pancreatic, and biliary organoids from both adult and pluripotent stem-cell sources [2, 3]. For transplantation, the appeal is concrete: donor scarcity, ischaemia–reperfusion injury and post-transplant complications remain inadequately addressed by available models, and human organoid systems now offer a tractable middle ground between cell lines and animal experimentation. We previously outlined an agenda for bringing organoid technology and organ transplantation into closer dialogue [4]. The contributions assembled in this Special Issue make clear how far that convergence has come, and what remains to be done.

What gives organoids their traction in transplant research is the combination of human cellular identity, three-dimensional architecture, and increasingly tractable manipulation through bioengineering [5]. These properties allow organoids to recapitulate developmental programmes, injury responses, and host–graft interactions in human tissue, and to do so with sufficient fidelity that organoid readouts can be compared directly against paired clinical specimens. Work over the past several years has also established that incorporating supporting cell types into composite organoids can improve viability, vascularisation, and engraftment after transplantation, providing a design principle that now informs cell-replacement strategies across organ systems [6–8].

The five papers in this issue articulate a shared trajectory: organoids are no longer being deployed merely as *in vitro* proxies for transplantation, but as entities prepared for it.

Shankar et al. address one of the most central questions for organoid transplantation: how human organoid tissue interacts with the immune system. Using human iPSC-derived kidney organoids exposed to peripheral blood mononuclear cells *in vitro* and after implantation in immune-deficient mice followed by human immune-cell transfer, the authors show that organoids are not immunologically inert. They undergo infiltration by T cells and macrophages, with stromal activation, induction of immune-response genes, epithelial PD-L1 expression, fibrosis-associated changes, and reduced expression of nephron differentiation and functional markers. This work provides an important platform for modelling alloimmune injury in human kidney-like tissue and emphasizes that immunogenicity must be integrated early into organoid translation.

Two papers focus on biliary complications after liver transplantation, a persistent clinical problem and a highly relevant test case for organoid-based modelling. Rejas and Junger provide a comprehensive review of cholangiocyte organoids in liver transplantation, highlighting their capacity to model cholangiopathies, ischemia–reperfusion injury, primary sclerosing cholangitis, primary biliary cholangitis, and genetic biliary disease. The review is particularly valuable in connecting organoid plasticity with clinically meaningful regeneration, including the possibility of using cholangiocyte organoids to repair injured bile ducts, support personalized medicine, and eventually reduce the burden of post-transplant cholangiopathy.

Complementing this conceptual synthesis, Kreiner et al. establish extrahepatic cholangiocyte organoids as an experimental model of ischemia–reperfusion injury during liver transplantation. By comparing organoids exposed to hypoxia and reoxygenation with human bile duct specimens obtained during cold storage and after reperfusion, the authors show that extrahepatic bile duct organoids recapitulate key features of biliary injury, including epithelial disruption, altered ZO-1 organization, hypoxic stress responses, ACSL4-associated ferroptosis signalling, apoptosis-related changes, and proliferative recovery after reoxygenation. This study provides a translationally anchored model for testing cholangio-protective interventions before clinical application.

Juksar et al. extend the discussion to pancreatic regeneration and beta-cell replacement. In adult human pancreatic ductal organoids, the authors examine how NEUROG3 induction influences endocrine differentiation and delamination, processes central to pancreatic development. They show that modulation of Notch, YAP, and EGFR pathways affects NEUROG3 expression, while doxycycline-induced NEUROG3 activates downstream endocrine programs and generates CHGA-positive endocrine progenitor-like cells. The addition of T3 and the AXL inhibitor R428 improves endocrine marker expression, including C-peptide, although full beta-cell identity remains incomplete. Importantly, the study documents delamination of NEUROG3-positive cells from the organoid epithelium, thereby modelling an essential developmental step in a human adult ductal system.

Finally, Montagud-Marrahi et al. bring organoids into the domain of organ preservation and targeted delivery. Using human and rat kidney tubuloids, they compare culture protocols and then evaluate delivery through direct injection versus normothermic machine perfusion (NMP). Their findings suggest that normothermic perfusion achieves broader and more efficient kidney targeting than local injection. In rat transplantation models, delivered tubuloids persisted, integrated into the host parenchyma, and formed tubular structures with a predominantly proximal phenotype; in a discarded human kidney, perfusion enabled cortical delivery of tubuloids and was associated with lower expression of selected injury and inflammatory markers. Although still preliminary, this work points to *ex vivo* perfusion as a clinically realistic bridge between organoid biology and graft-directed regenerative therapy.

Together, these contributions show that organoids are no longer only “mini-organs” for developmental biology. They are becoming experimental grafts, immune targets, disease avatars, injury sensors, drug-testing platforms, and vehicles for regeneration. The field now faces important challenges: improving maturation, vascularisation, innervation where relevant, scalability, reproducibility, genetic and epigenetic stability, immune compatibility, and regulatory standardisation. Equally important, ethical and societal reflection must accompany the science. The growing use of discarded human organs as preclinical platforms, the prospect of gene-edited or hypoimmune organoid products, and the unequal global access to advanced therapy medicinal products [9] each raise questions that the transplant community cannot leave to others to resolve.

This Special Issue captures a field in transition. Organoid technology will not replace transplantation in the immediate future, nor should its current limitations be underestimated. Yet these studies make clear that organoids are already reshaping how we understand graft injury, test interventions, and imagine regenerative solutions for organ failure. Their greatest contribution may be to transform transplantation research from the replacement of damaged organs alone toward the active repair, modelling, and engineering of transplantable tissues.
